# The Chronic Effect of Stair Climbing–Descending Exercises after Meals on Glycemic Control in Individuals with Type 2 Diabetes: A Randomized Controlled Trial

**DOI:** 10.3390/muscles2020018

**Published:** 2023-06-15

**Authors:** Hiroto Honda, Hiromi Fukutomi, Makoto Igaki, Shinichiro Tanaka, Tetsuo Takaishi, Tatsuya Hayashi

**Affiliations:** 1 Faculty of Rehabilitation, Shijonawate Gakuen University, Daito 574-0011, Japan; 2 Department of Rehabilitation, Toyooka Hospital Hidaka Medical Center, Toyooka 669-5392, Japan; 3 Department of Rehabilitation, Toyooka Hospital, Toyooka 668-8501, Japan; 4 Department of Internal Medicine, Toyooka Hospital Hidaka Medical Center, Toyooka 669-5392, Japan; 5 Graduate School of Science, Nagoya City University, Nagoya 467-8501, Japan; 6 Graduate School of Human and Environmental Studies, Kyoto University, Kyoto 606-8501, Japan

**Keywords:** physical activity, glycoalbumin, postprandial hyperglycemia, muscle strength

## Abstract

This study aimed to examine the chronic effect of a stair climbing–descending exercise (ST-EX) program on glycemic control in individuals with type 2 diabetes (T2D). Sixteen T2D participants were randomly divided into two groups and followed up over 12 weeks: they either performed regular ST-EX after meals at home (*n* = 8) or maintained their daily routine (CON; no training) (*n* = 8). The participants in the ST-EX group were instructed to perform a minimum of 12 sessions/week of ST-EX for more than three days/week. One session of ST-EX consisted of two repetitions of 3 min bouts of climbing to the second floor, followed by walking down to the first floor. Fourteen participants completed the study (seven for each group). The decrease in glycoalbumin levels was significantly greater in the ST-EX group (mean value: −1.0%) than in the CON group (+0.4%). Moreover, the knee extension force increased greatly in the ST-EX group (+0.2 Nm/kg) compared with that in the CON group (−0.1 Nm/kg), with no significant change in the skeletal muscle mass. Performing regular ST-EX after meals may be an effective strategy to improve glycemic control and lower-extremity muscle strength in individuals with T2D.

## 1. Introduction

Performing regular exercises, such as aerobic and resistance exercises, is important for the management of type 2 diabetes (T2D). Moderate-intensity aerobic exercise sessions of ≥10 min per bout for ≥150 min/week, or high-intensity exercise (HIE) for ≥75 min/week, have been recommended to individuals with T2D if the individuals are sufficiently fit [1]. Conversely, studies on whether short-duration HIE improves glycemic control found that repeated bouts of HIE could be equally or more effective than moderate-intensity exercises in reducing blood glucose (BG) levels [2,3]. Additionally, HIE for even short durations of <10 min (e.g., six bouts of 1 min high-intensity incline walking) could decrease postprandial BG levels and 24 h BG levels [3,4,5,6]. Furthermore, a recent randomized controlled trial showed the chronic effect of short-duration HIE on glycated hemoglobin (HbA1c) in individuals with T2D [7]. An eight-week HIE program (five bouts of two minutes of walking/running on a treadmill at 100% of VO_2_max with an interval of two minutes) significantly improved HbA1c levels (mean value: −1.80%). In that study, a continuous moderate-intensity walking/running program at 70% of VO_2_max for 14 min showed no significant change (−0.59%). However, the need for adaptation to physically fit patients, exercise equipment, and/or exercise facilities and a dependence on weather conditions are practical limitations of HIEs.

Previous studies reported on the effects of stair climbing–descending exercise (ST-EX), which is a short-duration HIE modality, on postprandial and 24 h BG excursions in individuals with T2D [8,9]. ST-EX comprises one or two repetitions (3 min per bout) of exercises using stairs: climbing to the second floor (80–110 steps/min) followed by walking down to the first floor at a free step rate. This is a time-saving and useful HIE, regardless of weather conditions, training clothes, and access to an exercise facility. Furthermore, most patients can perform ST-EX without serious symptoms, such as dyspnea or leg exhaustion [8].

However, whether a long-term ST-EX intervention has a chronic effect on glycemic control, including HbA1c, remains unclear. A previous study examined the effect of a 2-week ST-EX program that comprised repeated 3 min ST-EX starting 60 and 120 min after each meal on the serum 1,5-anhydroglucitol (1,5-AG) levels of individuals with T2D and found that the program significantly increased their 1,5-AG levels after the exercise period [10]. The 1,5-AG level is closely associated with BG fluctuations and reflects the 2 h postprandial BG level in the two preceding weeks in individuals with T2D [11]. Therefore, a longer-term ST-EX program may also have a chronic effect on glycemic control via improvements in postprandial and persistent hyperglycemia [8,9,10]. This study aimed to examine the chronic effect of a 12-week ST-EX program on glycemic control in individuals with T2D.

## 2. Results

Of the 16 participants who were enrolled in this study and randomized into the two groups, 2 participants dropped out and the remaining 14 completed this study (7 for each group). The baseline characteristics of the analyzed participants are shown in Table 1. No significant differences were found between the ST-EX (performing regular ST-EX) and CON (no training) groups for all the variables. Although two participants had occasionally exercised before baseline, they were not placed in the category of having daily exercise habits (≥30 min/day, ≥2 times/week, and ≥1 year [12]). Additionally, nutritional therapy and medication in the participants remained stable with no major changes throughout the study period.

The median (lower and upper quartiles) frequency of ST-EX was 11.8 (7.2, 15.5) sessions and 6.5 (5.3, 7.0) days per week, and the proportion of participants who were fully adherent (completed ≥12 sessions/week and three days/week for 12 weeks) was 43% (three out of seven). Regarding each time point, the participants performed ST-EX most frequently 60 min after breakfast (median number of ST-EX sessions: breakfast, 60 min: 5.5 sessions/week, 120 min: 0.5 sessions/week; lunch, 60 min: 2.3 sessions/week, 120 min: 0.4 sessions/week; dinner, 60 min: 4.6 sessions/week, and 120 min: 0.2 sessions/week).

**Figure 1:**
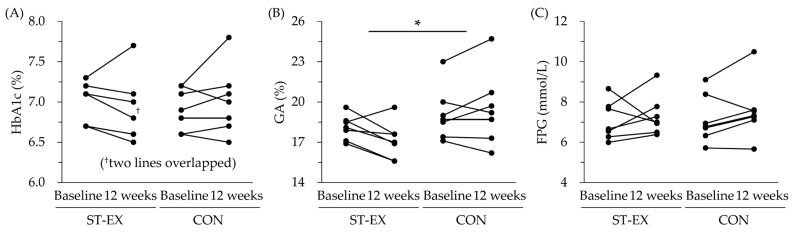
Changes in glycemic control in each participant from baseline to 12 weeks later in the stair climbing–descending exercise (ST-EX) group and no training (CON) group. (**A**) Glycated hemoglobin (HbA1c). (**B**) Glycoalbumin (GA). (**C**) Fasting plasma glucose (FPG). * *p* < 0.05, comparison of change between ST-EX group (*n* = 7) and CON group (*n* = 7), unpaired *t*-test.

The changes in glycemic control in each participant are shown in Figure 1. The HbA1c and glycoalbumin (GA) values of each participant declined in the ST-EX group, except in the participant who performed ST-EX the least frequently (7.1 sessions/week). The changes in glycemic control and metabolic variables (homeostasis model assessment of insulin resistance, blood pressure, and serum lipids) between baseline and 12 weeks are shown in Table 2. The decreases in GA level were greater in the ST-EX group than in the CON group (*p* = 0.037), and the amount of changes in their other glycemic parameters and metabolic function did not differ significantly between the groups.

With respect to body composition, motor function, and physical activity (PA), only knee extension force increased to a greater degree in the ST-EX group than in the CON group (*p* = 0.032) (Table 3): the mean rate of increase from baseline in the ST-EX group was 21.9%. Conversely, no significant difference was observed in the skeletal muscle mass index (SMI), although that tended to be stable in the ST-EX group and decreased in the CON group.

## 3. Discussion

The 12-week ST-EX program improved GA levels and knee extension force in individuals with T2D. The findings contribute to the self-management of diabetes, as ST-EX is a time-efficient short-duration HIE that can be performed indoors by patients at home at a self-directed intensity. In addition, ST-EX requires no specific sportswear or equipment and is cost-free. A recent systematic review and meta-analysis showed that HIE was more effective than moderate-intensity exercise for glucose metabolism management even in older people, especially with the benefit of being time-efficient [13].

As mentioned earlier, 3 min ST-EX performed after meals has an acute effect on postprandial and 24 h BG excursions in individuals with T2D [8,9]. HIE can increase the rate of glucose uptake in skeletal muscles, which reaches a near-maximal level within 10 min after the start of exercise [14], depending on the exercise intensity [15,16]. Factors that acutely increase glucose uptake include enhancements in the translocation of glucose transporter 4 and the delivery of glucose and insulin during and immediately after exercises via improvements in endothelial function [17]. In this study, the participants in the ST-EX group continued exercising regularly during the study period, which could modulate the acute increase seen in BG levels after meals. Hence, GA levels, which are a more sensitive parameter for postprandial hyperglycemia than HbA1c levels [18,19], may improve after completing the 12-week ST-EX program. A previous study showed that a 12-week supervised aerobic (30 min) and resistance exercise program performed three times/week, gradually increasing in intensity throughout the study period, improved GA levels by −1.5% compared with standard therapy [20]. Matching the decreased GA levels reported in the previous study, the current study found that GA levels decreased even after an unsupervised brief bout of exercise (ST-EX: −1.0%, CON: +0.4%). Therefore, completing the ST-EX protocol for 12 weeks is considered a useful method for improving GA levels in individuals with T2D.

GA levels reflect short-term (2–3 weeks) mean BG levels in individuals with T2D [21]. A GA assessment is helpful for clinicians to evaluate early treatment responses in glycemic control and whether the treatment can improve future HbA1c levels [22], which reflects average BG levels over approximately 3 months [23]. In this study, the 12-week ST-EX program caused a decrease in GA levels but not a significant change in HbA1c levels. Thus, a longer period than the one used in this study (e.g., 16, 20, or 24 weeks) may improve HbA1c levels. Furthermore, HbA1c levels may change if ST-EX adherence (e.g., more frequent monitoring and feedback [24]) improves and intensity incrementally increases as the muscle strength of individuals improves with exercise.

The target frequency was set at a minimum of 12 sessions/week of ST-EX such that the net exercise time per week (72 min/week) became approximately the same as the volume of HIE recommended (minimum: 75 min/week) [1]. As a result, the median volume was 70.8 min/week (6 min/session (net exercise time) × 11.8 sessions/week), and 57% of participants (four out of seven) did not perform the instructed sessions. Therefore, the chronic effect of ST-EX on glycemic control may even be underestimated in this study, affected by the lack of exercise volume and adherence. Conversely, although subjectively assessed PA increased slightly in the ST-EX group 12 weeks later, no difference between the groups was observed. Therefore, the effect obtained in this study might be induced not simply by increased PA, but rather by performing regular ST-EX. 

Changes in other metabolic functions and body composition did not differ between the groups. Body weight; waist circumference, reflective of the amount of visceral fat; and fat mass changes following exercise may be affected by activity volume and energy expenditure [25,26]. Additionally, although low-volume HIE can improve lipid profiles and insulin sensitivity, it may not be superior to moderate-intensity continuous exercise and high-volume HIE [27,28,29]. It could be assumed that this study protocol may be insufficient to improve these parameters, due to the low exercise volume and lack of supervision during exercise [30,31,32]. Furthermore, the lack of changes after the intervention might be due to the levels of those parameters being nearly within the normal range (e.g., office blood pressure < 140 mmHg, triglycerides < 150 mg/dL, and low-density lipoprotein cholesterol < 140 mg/dL) at baseline in both groups [33,34,35].

The motor functions of the participants were sufficient, and no participants were diagnosed with sarcopenia (SMI: <7.0 kg/m^2^ in males and <5.7 kg/m^2^ in females; grip strength: <28 kg in males and < 18 kg in females; 5-repetition sit-to-stand test (5R-STS): ≥12 s; and gait speed: <1.0 m/s, respectively) [36]; thus, changes in parameters related to sarcopenia were not observed in this study. Conversely, their knee extension force significantly increased after 12 weeks. Regarding the stair exercise, its effect on the muscle strength of lower extremities remains unclear. A previous study reported that 42 steps × three sets per day of stair exercises while wearing a weighted vest with 2 min of intervals between sets three days/week induced a 9.6% increase in knee extension strength (N/kg) after 12 weeks in older adults [37]. Another study on step exercises (not stairs climbing) found that four sets of 10 step-up repetitions × three times per day of step exercise while wearing a weighted vest three days/week induced an increase, although not significantly, in knee extension strength (Nm) of 9.2% in older women after six weeks [38]. The larger volume of steps performed weekly during ST-EX in this study compared with that in previous studies may have contributed to the larger increase in knee extension force (21.9% increase from baseline).

In this study, no adverse events (e.g., orthopedic disorders) occurred during the study period. Additionally, no falls or slips occurred on the stairs, despite the age of the participants ranging from 50 to 74 years, within which age range falls on stairs among older individuals are common. Deteriorations in physical function, such as low muscle strength due to diabetic neuropathy, may also increase the risk of accidents during stair exercises, especially during descent [39]. Therefore, the risk of falling should be considered (e.g., by using handrails) when older patients and/or patients with complications of diabetes or physical dysfunction (e.g., motor disorders) perform exercises using stairs.

The biggest strength of this study is that this is the first study to prove the chronic effect of exercise using stairs in individuals with T2D. ST-EX is a new effective exercise modality that is self-managed and can be performed regularly wherever stairs are nearby (e.g., in the office), even by patients who cannot perform generally recommended exercises [1] due to various difficulties, such as a lack of time and equipment. However, this study has some weaknesses. First, this study was a single-center open-label randomized controlled trial and had a small sample size of participants with uncomplicated diabetes, since many patients declined to participate due to various reasons. Second, the amount of PA other than ST-EX that participants engaged in was not objectively measured. Additionally, the ST-EX program in this study was not compared with other exercise modalities, although a previous study found that a single bout of ST-EX for 6–6.5 min decreased postprandial BG levels, compared with a walking exercise for the same duration in individuals with T2D [40].

## 4. Materials and Methods

### 4.1. Participants

**Figure 2:**
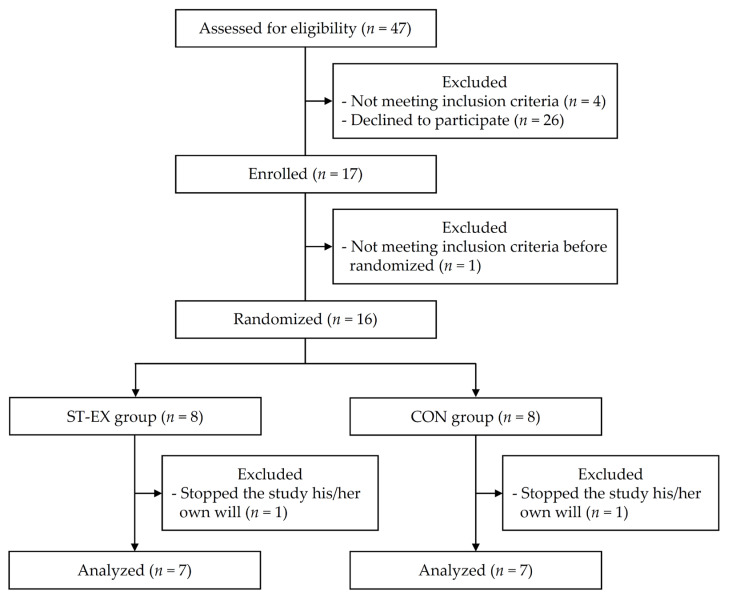
Flow chart of study population selection, including patient recruitment. ST-EX: stair climbing–descending exercise; CON: no training.

The study period was between September and December of 2021 (12 weeks). A total of 37 adults with uncomplicated T2D aged ≤74 years who regularly visited the Toyooka Hospital Hidaka Medical Center (Toyooka, Japan) as outpatients were assessed according to the exclusion criteria: diabetes diagnosed less than a year ago, restricted PA due to cardiovascular or motor dysfunction, severe chronic complications of diabetes (e.g., diabetic retinopathy) and/or cognitive decline, and no appropriate stairs in their living environments. A sample size of 34 participants (17 per group), with an attrition rate of 10%, was considered sufficient based on a previous study (*α* value: 0.05; power: 80%; and effect size: 0.8) [41]. Although patient recruitment aimed for a total of 34 participants, many patients did not agree to participate in this study due to reasons such as a lack of time and/or interest in exercise. Ultimately, the number of participants fell short of 34, and only 17 participants aged 50–74 years enrolled in this study. Additionally, after written informed consent was obtained from all 17 participants, 1 participant was excluded due to not meeting the criteria after consenting. Finally, 16 participants were randomly allocated at a 1:1 ratio to the ST-EX (*n* = 8) and CON (*n* = 8) groups with stratification by sex (Figure 2) via random number assignment generated by a computer. No participants or investigators were blinded to the group assignments.

All participants received medical nutritional education (energy intake: 25–30 kcal/kg/day) and consulted dieticians once every 1–2 months. Thirteen (ST-EX: 6 participants, CON: 7 participants) of sixteen participants took oral hypoglycemic agents, and their conditions remained stable, with no major changes (e.g., from oral hypoglycemic agents to insulin injections) six months before the baseline (September of 2021). No participant took β-blockers, which could have affected their heart rate responses to exercise.

### 4.2. Study Design

This was an open-label randomized controlled trial that was registered with the UMIN Clinical Trials Registry (UMIN000042972). The protocol was approved by the institutional review board of Shijonawate Gakuen University (approval number: 21-11) in accordance with the Declaration of Helsinki.

### 4.3. Exercise Protocol

In the ST-EX group, participants were instructed by a physical therapist to perform one session of ST-EX in their homes starting both or either 60 or 120 min after their meals, according to previous study protocols [8,9,10], and to perform at least 12 sessions of ST-EX per week for more than three days per week for 12 weeks (from September to December of 2021). One session of ST-EX consisted of two repetitions of 3 min bouts of climbing and descending the stairs between the first and second floors (11–15 steps up; each 17–23 cm in height; 4–7 round trips; 104–156 steps/bout), with a 1–2 min interval between the bouts. The total duration per session was 7–8 min, and the total volume per week was 84–96 min with intervals (net exercise time per week: 72 min). Participants could choose to use handrails during their exercise. At baseline, the participants were instructed on how to perform ST-EX. Within a 3 min bout, the participants increased the intensity of ST-EX up to 70–80% of their age-predicted maximum heart rate, checking their heart rate every minute using a heart rate monitor (Polar Accurex Plus monitor; Polar Electro, Kempele, Finland). Climbing speed at that intensity was within the range of 80–110 steps/min [8]. Descending speed was set at a free step rate; thus, subjective intensity during ST-EX was within the range of moderate (Borg rating of perceived exertion (RPE) score [42], 12–13) [8]. 

Apart from performing ST-EX, both groups were asked to continue their daily routines (e.g., occupational and leisure activities) but were not allowed to perform any new exercises throughout the observation period. PA changes from baseline to the end of observation were evaluated using the questionnaire described below. Only the participants in the ST-EX group underwent follow-ups via telephone every four weeks (four and eight weeks after baseline) to check for adherence and adverse events to the exercise program.

### 4.4. Measurements

The primary outcome was changes in the HbA1c and GA levels from baseline to 12 weeks. The secondary outcome was changes in other metabolic functions, body composition, motor function, and PA. All variables were measured the day before the start and after the end of the program in the morning after an overnight fast. Participants were told not to drink beverages containing caffeine or alcohol or to smoke before measurements were taken. Venous blood samples were collected to measure blood parameters. HbA1c levels were determined using a high-performance liquid chromatographic method. Other parameters (GA, BG, and serum lipids levels) were measured using enzymatic or direct methods.

Body fat and skeletal muscle mass were measured via bioelectrical impedance analysis using InBody S10 (InBody Japan Inc., Tokyo, Japan) in the standing position, and SMI was calculated by dividing upper and lower limb muscle mass by their squared height. Grip strength was measured using a Smedley-type grip-strength meter (TKK 5401; Takei Scientific Instruments Co., Ltd., Niigata, Japan) in the standing position. The tested upper extremity was positioned by the side of the body with the elbow extended and the handle of the dynamometer resting in the middle of four fingers. Knee extension strength (maximal isometric strength) was measured using a handheld dynamometer with a fixation belt (μTas F-1; Anima Corp., Tokyo, Japan) in the sitting position with the hip and knee joints bent at 90° and the arms folded across the chest. In both strength assessments, the mean strength values were calculated using the maximum values of two measurements carried out on each limb. Regarding knee extension strength, the force (Nm/kg) was calculated by multiplying the measured values by the length of the lower leg and dividing by the body weight. The 5R-STS test was performed following instructions to rise as quickly as possible from a seated position to a standing posture while keeping the arms folded across the chest, measured using a stopwatch, and the time from the initial command to the fifth completed stand was recorded. Gait speed was assessed via the 5 m gait speed test using a stopwatch, and the participants were instructed to walk at their comfortable/maximum pace from a few steps before the start line to a few steps passed the 5 m mark. For both the 5R-STS and gait speed tests, the mean values of two measurements were calculated. Participants’ PA was assessed using the International Physical Activity Questionnaire, with a recall period of the last seven days [43]. Furthermore, the number of ST-EX sessions throughout the observation period was self-reported using a recording sheet.

### 4.5. Statistical Analysis

All values were reported as the mean (95% confidence interval) or median (lower and upper quartiles). The normality of the distributions was checked using a Shapiro–Wilk test. The variables at baseline in the ST-EX and CON groups were compared using an unpaired *t*-test or Mann–Whitney U test for continuous variables and Fisher’s exact test for nominal variables. The change in each variable from baseline to 12 weeks in both groups was compared using an unpaired *t*-test or Mann–Whitney U test. All analyses were performed using IBM SPSS Statistics (version 27.0; IBM Corp., Armonk, NY, USA). Statistical significance was set at *p* < 0.05.

## 5. Conclusions

Regularly exercising using stairs for 12 weeks improved the glycoalbumin levels and knee extension force of individuals with type 2 diabetes. This method of exercise is a time-efficient and easy-to-perform high-intensity indoor exercise even if patients have no exercise equipment. Although this study had an insufficient sample size and did not show comparisons with other exercise modalities, the presented stair exercise program may be a useful method of managing glycemic control, as well as motor function, in patients with type 2 diabetes.

## Figures and Tables

**Table 1:** Characteristics of the participants analyzed at baseline.

Variables	ST-EX (*n* = 7)	CON (*n* = 7)	*p*-Value
Sex (female) (*n*) (%)	3 (42.9)	4 (57.1)	1.000
Age (years)	71.0 (59.0, 73.0) ^†^	69.0 (63.0, 71.0) ^†^	0.383
Duration of diabetes (years)	11.0 (7.1, 14.9)	9.9 (5.1, 14.6)	0.657
HbA1c (%) (mmol/mol)	7.0 (6.7, 7.2) (52.7 (50.0, 55.3))	6.9 (6.6, 7.2) (52.1 (49.4, 54.7))	0.690
GA (%)	18.1 (17.2, 19.0)	19.1 (17.3, 20.9)	0.249
FPG (mmol/L)	7.1 (6.2, 8.0)	7.1 (6.0, 8.2)	0.925
BMI (kg/m^2^)	24.4 (22.5, 26.3)	23.8 (20.0, 27.5)	0.711
Medication			
Biguanide (*n*) (%)	1 (14.3)	4 (57.1)	0.266
Thiazolidine (*n*) (%)	0 (0.0)	1 (14.3)	1.000
α-glucosidase inhibitor (*n*) (%)	0 (0.0)	4 (57.1)	0.070
Sodium-glucose cotransporter-2 inhibitor (*n*) (%)	1 (14.3)	2 (28.6)	1.000
Dipeptidyl peptidase-4 inhibitors (*n*) (%)	2 (28.6)	1 (14.3)	1.000
Sulfonylurea (*n*) (%)	1 (14.3)	3 (42.9)	0.559
Hypertension (*n*) (%)	4 (57.1)	4 (57.1)	1.000
Dyslipidemia (*n*) (%)	4 (57.1)	2 (28.6)	0.592
Cigarette smoking ^‡1^			0.559
Non-smoker (*n*) (%)	4 (57.1)	5 (71.4)	
Past smoker (*n*) (%)	3 (42.9)	1 (14.3)	
Current smoker (*n*) (%)	0 (0.0)	1 (14.3)	
Alcohol drinking (*n*) (%) ^‡2^	2 (28.6)	1 (14.3)	1.000
Exercise habits (*n*) (%) ^‡3^	0 (0.0)	0 (0.0)	–
Occupation			1.000
Unemployed (*n*) (%)	3 (42.9)	4 (57.1)	
Farmer (*n*) (%)	2 (28.6)	1 (14.3)	
Sedentary worker (*n*) (%)	1 (14.3)	1 (14.3)	
Other (*n*) (%)	1 (14.3)	1 (14.3)	

Values are presented as the mean (95% confidence interval), median (lower and upper quartiles) (^†^), or number (percentage). ST-EX: stair climbing–descending exercise; CON: no training; HbA1c: glycated hemoglobin; GA: glycoalbumin; FPG: fasting plasma glucose; BMI: body mass index. ^‡1^ ≥1 time/day. ^‡2^ ≥30 g/day for male participants and ≥20 g/day for female, ^‡3^ ≥30 min/day, ≥2 times/week, ≥1 year. *p*-value: ST-EX group vs. CON group, unpaired *t*-test, Mann–Whitney U test (^†^), or Fisher’s exact test.

**Table 2:** Comparison of glycemic control and metabolic variables between baseline and 12 weeks (^†^: median value).

Variables	Group	Baseline	12 Weeks	Change	Effect Size (Cohen’s *d*, *r*)	*p*-Value
HbA1c (%) (mmol/mol)	ST-EX	7.0 (6.7, 7.2) (52.7 (50.0, 55.3))	6.9 (6.5, 7.3) (51.9 (47.7, 56.1))	−0.1 (−0.2, −0.1) (−1.1 (−2.2, −1.1)) ^†^	0.420	0.128
	CON	6.9 (6.6, 7.2) (52.1 (49.4, 54.7))	7.0 (6.6, 7.4) (53.1 (48.9, 57.4))	0.1 (−0.1, 0.2) (1.1 (−1.1, 2.2)) ^†^		
GA (%)	ST-EX	18.1 (17.2, 19.0)	17.1 (15.9, 18.4)	−1.0 (−2.0, 0.0)	1.257	0.037 *
	CON	19.1 (17.3, 20.9)	19.5 (17.0, 22.0)	0.4 (−0.6, 1.4)		
FPG (mmol/L)	ST-EX	7.1 (6.2, 8.0)	7.3 (6.4, 8.2)	0.2 (−0.8, 1.3)	0.221	0.687
	CON	7.1 (6.0, 8.2)	7.6 (6.2, 8.9)	0.4 (−0.2, 1.1)		
FIRI (pmol/L)	ST-EX	38.4 (24.6, 67.2) ^†^	33.0 (21.6, 59.4) ^†^	−3.0 (−7.8, 1.2) ^†^	0.308	0.259
	CON	39.0 (19.2, 51.6) ^†^	39.0 (19.2, 46.2) ^†^	0.0 (−4.2, 9.6) ^†^		
HOMA-IR	ST-EX	1.8 (1.2, 3.1) ^†^	1.8 (1.4, 2.6) ^†^	0.0 (−0.8, 0.2) ^†^	0.290	0.318
	CON	1.7 (1.2, 3.2) ^†^	1.9 (1.5, 2.6) ^†^	0.1 (0.0, 0.7) ^†^		
SBP (mmHg)	ST-EX	131.0 (114.0, 138.0) ^†^	138.0 (121.0, 139.0) ^†^	3.0 (−2.7, 8.7)	0.239	0.663
	CON	125.0 (111.0, 136.0) ^†^	128.0 (124.0, 133.0) ^†^	5.4 (−6.6, 17.5)		
DBP (mmHg)	ST-EX	74.9 (65.5, 84.3)	74.6 (65.8, 83.3)	−0.3 (−4.5, 3.9)	0.093	0.864
	CON	73.6 (61.9, 85.3)	72.7 (62.8, 82.6)	−0.9 (−7.7, 6.0)		
TG (mmol/L)	ST-EX	1.7 (0.6, 2.6) ^†^	1.3 (0.6, 2.4) ^†^	−0.2 (−0.5, 0.1)	0.294	0.592
	CON	1.2 (0.9, 1.6) ^†^	0.9 (0.8, 1.7) ^†^	−0.1 (−0.5, 0.4)		
LDL-C (mmol/L)	ST-EX	2.9 (2.1, 3.6)	2.8 (2.3, 3.4)	0.0 (−0.4, 0.3)	0.309	0.574
	CON	3.0 (2.5, 3.6)	2.9 (2.0, 3.7)	−0.2 (−0.6, 0.3)		
HDL-C (mmol/L)	ST-EX	1.4 (1.1, 1.6) ^†^	1.6 (1.4, 1.7) ^†^	0.1 (−0.1, 0.2)	0.096	0.861
	CON	1.4 (1.3, 1.9) ^†^	1.5 (1.4, 1.7) ^†^	0.1 (−0.1, 0.3)		
Non-HDL-C (mmol/L)	ST-EX	3.6 (2.8, 4.5)	3.5 (3.0, 4.0)	−0.1 (−0.6, 0.3)	0.166	0.761
	CON	3.6 (3.0, 4.2)	3.4 (2.7, 4.1)	−0.2 (−0.6, 0.1)		

Values are presented as the mean (95% confidence interval) or median (lower and upper quartiles) (^†^). HbA1c: glycated hemoglobin; ST-EX: stair climbing–descending exercise; CON: no training; GA: glycoalbumin; FPG: fasting plasma glucose; FIRI: fasting immunoreactive insulin; HOMA-IR: homeostasis model assessment of insulin resistance; SBP: systolic blood pressure; DBP: diastolic blood pressure; TG: triglyceride; LDL-C: low-density lipoprotein cholesterol; HDL-C: high-density lipoprotein cholesterol. *p*-value: comparison of change between ST-EX group (*n* = 7) and CON group (*n* = 7), unpaired *t*-test or Mann–Whitney U test (^†^). * *p* < 0.05.

**Table 3:** Comparison of body composition, motor function, and PA between baseline and 12 weeks (^†^: median value).

Variables	Group	Baseline	12 Weeks	Change	Effect Size (Cohen’s *d*, *r*)	*p*-Value
Body weight (kg)	ST-EX	63.6 (57.7, 69.5)	63.3 (57.9, 68.7)	−0.3 (−1.4, 0.8)	0.285	0.604
	CON	60.6 (48.5, 72.6)	60.0 (48.5, 71.5)	−0.6 (−1.5, 0.3)		
BMI (kg/m^2^)	ST-EX	24.4 (22.5, 26.3)	24.3 (22.5, 26.1)	−0.1 (−0.5, 0.3)	0.327	0.552
	CON	23.8 (20.0, 27.5)	23.5 (20.1, 27.0)	−0.2 (−0.6, 0.1)		
Waist circumference (cm)	ST-EX	89.1 (84.1, 94.2)	88.9 (82.7, 95.0)	−2.0 (−2.0, 0.5) ^†^	0.155	0.620
	CON	88.8 (80.2, 97.4)	87.6 (79.3, 95.9)	−0.5 (−4.0, 1.0) ^†^		
Body fat mass (kg)	ST-EX	18.9 (15.8, 21.9)	19.2 (15.8, 22.6)	0.3 (−0.6, 1.4)	0.072	0.895
	CON	18.2 (10.4, 26.1)	18.5 (11.5, 25.4)	0.3 (−0.9, 1.4)		
Lower SMM (kg)	ST-EX	14.3 (12.7, 15.8)	14.3 (12.5, 16.1)	0.0 (−0.6, 0.6)	0.941	0.104
	CON	12.3 (9.6, 14.9)	11.8 (9.2, 14.4)	−0.5 (−0.8, −0.1)		
SMI (kg/m^2^)	ST-EX	7.2 (6.6, 7.9)	7.2 (6.5, 8.0)	0.0 (−0.2, 0.3)	0.945	0.102
	CON	6.5 (5.6, 7.3)	6.3 (5.5, 7.2)	−0.2 (−0.3, −0.1)		
Grip strength (kg)	ST-EX	25.8 (20.7, 35.7) ^†^	28.7 (22.9, 36.3) ^†^	1.7 (−0.5, 3.9)	0.199	0.716
	CON	21.3 (15.2, 27.3) ^†^	23.2 (17.7, 26.2) ^†^	1.3 (−0.3, 2.8)		
Knee extension force (Nm/kg)	ST-EX	0.9 (0.6, 1.3)	1.1 (0.8, 1.5)	0.2 (0.1, 0.3)	1.294	0.032 *
	CON	1.0 (0.7, 1.3)	0.9 (0.6, 1.2)	−0.1 (−0.3, 0.1)		
5R-STS test (s)	ST-EX	11.5 (7.5, 15.5)	9.0 (7.5, 10.5)	−0.4 (−5.8, 0.5) ^†^	0.188	0.535
	CON	8.9 (7.1, 10.7)	8.1 (6.8, 9.3)	−1.3 (−2.3, 1.3) ^†^		
Comfortable gait speed (m/s)	ST-EX	1.3 (1.1, 1.5)	1.3 (1.1, 1.5)	0.0 (−0.2, 0.1)	0.546	0.327
	CON	1.4 (1.2, 1.6)	1.4 (1.3, 1.6)	0.1 (−0.1, 0.2)		
Maximum gait speed (m/s)	ST-EX	1.9 (1.3, 2.4)	2.0 (1.6, 2.3)	0.1 (−0.3, 0.5)	0.002	0.996
	CON	1.7 (1.5, 2.0)	1.8 (1.7, 2.0)	0.1 (−0.1, 0.3)		
Total daily MVPA (METs-min/day)	ST-EX	257.5 (54.0, 460.9)	322.4 (110.6, 534.3)	65.0 (−70.8, 200.7)	0.215	0.695
	CON	336.1 (1.3, 673.5)	322.1 (153.6, 490.6)	−14.0 (−475.8, 447.8)		
Total daily EE in MVPA (kcal/day)	ST-EX	220.9 (62.5, 921.7) ^†^	271.3 (181.9, 494.9) ^†^	76.7 (−79.9, 233.2)	0.274	0.618
	CON	240.7 (65.0, 560.0) ^†^	259.1 (105.7, 634.1) ^†^	−27.1 (−497.5, 443.3)		
Sedentary behavior (min/day)	ST-EX	240.0 (120.0, 420.0) ^†^	240.0 (150.0, 420.0) ^†^	30.0 (−180.0, 90.0) ^†^	0.137	0.620
	CON	271.3 (181.9, 494.9) ^†^	360.0 (180.0, 480.0) ^†^	60.0 (−150.0, 120.0) ^†^		

Values are presented as the mean (95% confidence interval) or median (lower and upper quartiles) (^†^). ST-EX: stair climbing–descending exercise; CON: no training; BMI: body mass index; SMM: skeletal muscle mass; SMI: skeletal muscle mass index; 5R-STS: 5-repetition sit-to-stand; MVPA: moderate- to vigorous-intensity physical activity; EE: energy expenditure; *p*-value: comparison of change between ST-EX group (*n* = 7) and CON group (*n* = 7), unpaired *t*-test or Mann–Whitney U test (^†^). * *p* < 0.05.

## Data Availability

All relevant data are contained within the paper.
